# Tuberculous Extensor Tenosynovitis Presenting as Huge Mass on the Dorsum of the Hand

**DOI:** 10.7759/cureus.10236

**Published:** 2020-09-03

**Authors:** Oussama Mansour, Mohamad K Moussa, Zaynab Khalaf, Hussein Zreik, Ryan Bou Raad

**Affiliations:** 1 Orthopedics and Traumatology, Al Zahraa Hospital, University Medical Center, Beirut, LBN; 2 Orthopedic Surgery, Lebanese University Faculty of Medical Science, Beirut, LBN; 3 Endocrinology, Diabetes and Metabolism, Lebanese University Faculty of Medical Science, Beirut, LBN; 4 Orthopaedics and Trauma, Al Zahraa Hospital, University Medical Center, Beirut, LBN; 5 Orthopaedics and Traumatology, Lebanese University Faculty of Medicine, Beirut, LBN

**Keywords:** tuberculosis, infectious tenosynovitis, extensor tenosynovitis, hand

## Abstract

Infectious tuberculous tenosynovitis (TS) of the extensor tendons of the wrist is an exceptional location of musculoskeletal tuberculosis. We present a case of tuberculous extensor TS in a 52-year-old diabetic male patient presenting as a huge mass on the dorsum of the hand, in the absence of other pulmonary or extrapulmonary manifestation of tuberculosis. This report increases physicians’ vigilance when dealing with patients with risk factors of tuberculosis, allowing early diagnosis and optimal treatment.

## Introduction

Tenosynovitis (TS) is a type of tendinopathy resulting from idiopathic, infectious or noninfectious etiologies such as autoimmune, traumatic or mechanical problems [1]. Infectious TS is most commonly caused by *Staphylococcus aureus* and the site of involvement is usually the flexor tendons [2]. Tuberculous infectious TS of the extensor tendon is very rare in literature and has been mentioned in very few case reports [3,4]. We present herein a rare case of extrapulmonary manifestation of tuberculosis involving the hand extensor tendon sheath, presenting as huge mass on the dorsum of the hand, in the absence of any other signs or symptoms of tuberculosis.

## Case presentation

This is a 50-year-old male patient, known to have diabetes mellitus and hypercholesterolemia, working in a travel agency that provides pilgrimage services, presenting with two months’ history of painful edema on the dorsum of the right hand. He first sought medical advice from an infectologist who made a diagnosis of cellulitis that has failed treatment with amoxicillin-clavulanic acid.

**Figure 1:**
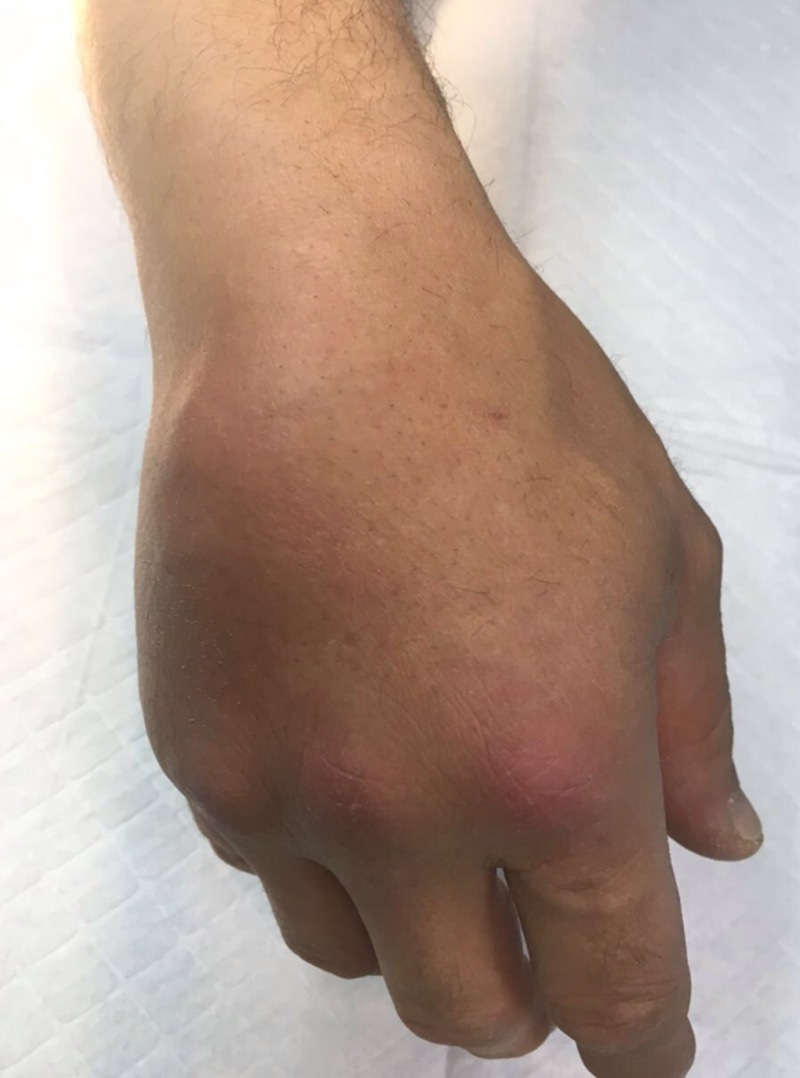
Mass-like cystic enlargement on the dorsum of the hand with limited extension of the 2nd till 5th finger and drop of the ulnar three digits

Upon physical examination, the patient had a tender fusiform edematous lesion on the dorsum of the hand with erythema extending to the metacarpophalangeal joints of the 2nd to the 5th fingers. On palpation, we felt a mass-like cystic enlargement on the dorsum of the hand with limited extension of the 2nd till the 5th fingers and a drop of the ulnar three digits (Figure 1). The patient was afebrile, without any other signs or symptoms.

Laboratory examinations including complete blood count, C-reactive protein (CRP), erythrocyte sedimentation rate (ESR), fasting blood sugar, and creatinine were all within normal limits. PPD test was negative. Plain radiographs of the hand were without any bony abnormal findings.

**Figure 2:**
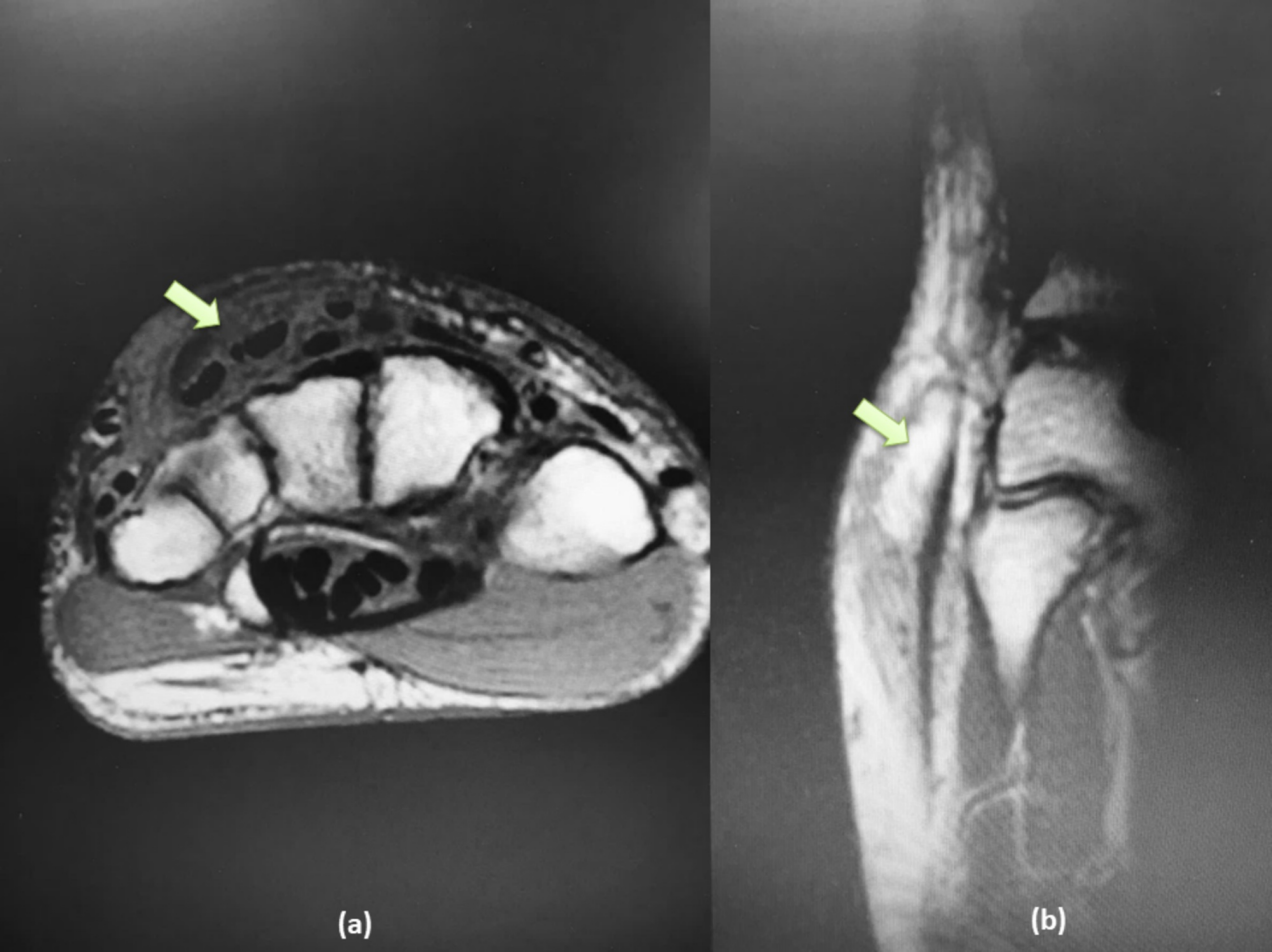
(a) Axial T1-weighted MRI cut showing well circumscribed mass circumferentially enclosing the extensors tendons of the hand, (b) sagittal T2-weighted images showing the extent of the mass

An MRI of the hand was ordered and revealed a well-circumscribed mass circumferentially enclosing the extensors tendons of the hand, compatible with a diagnosis of hypertrophic synovitis of the tendon sheath of the extensors (Figure 2).

**Figure 3:**
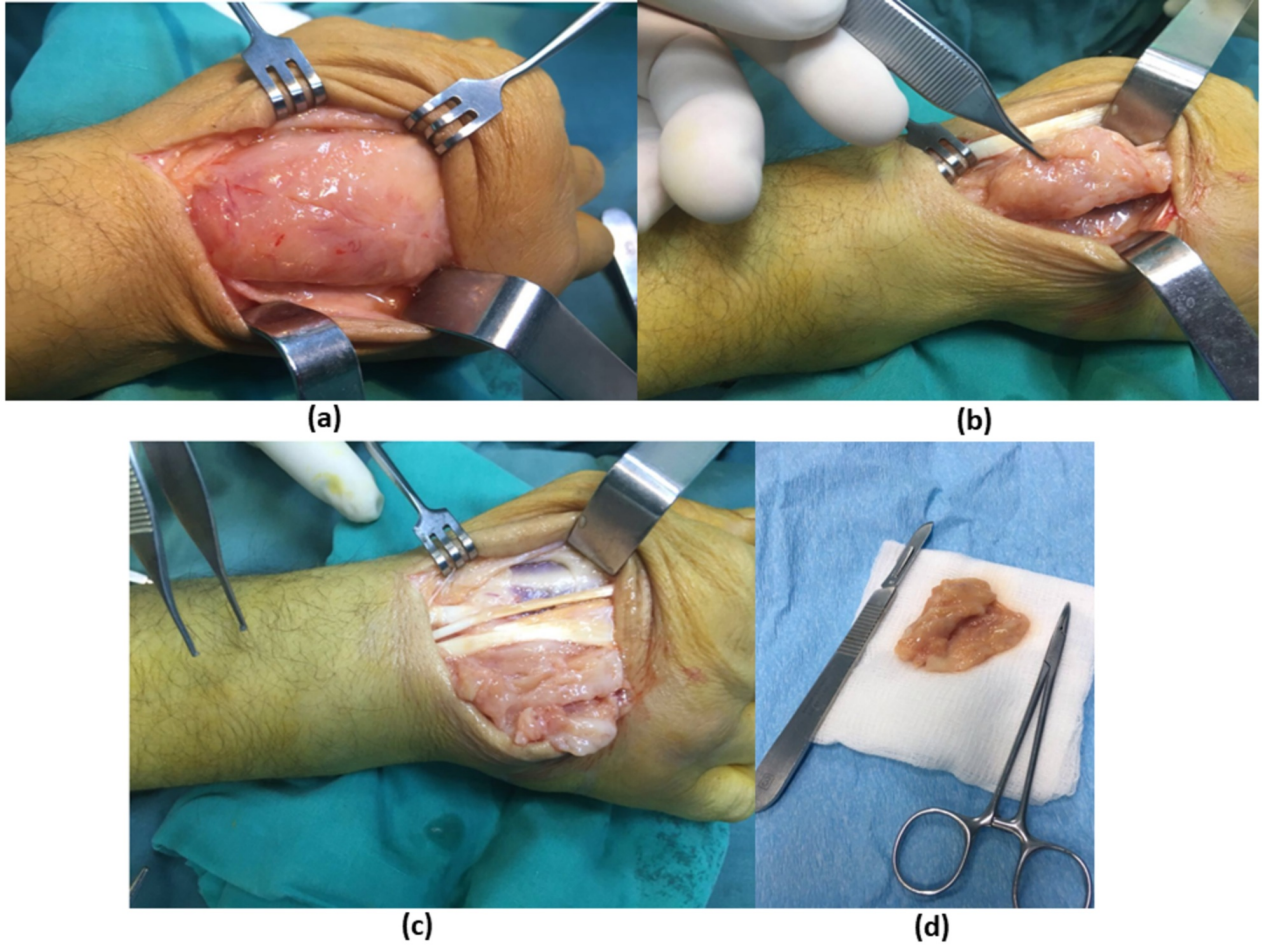
Different aspect of the surgical intervention: (a) mass which was completely adherent to the extensor tendons, (b) dissection to liberate the extensor tendons, (c) en bloc excision of the mass and (d) the mass isolated

The decision was made to address the pathology by surgical tenosynovectomy with specimens taken for culture and pathology. Under loco-regional anesthesia and the use of a tourniquet to secure blood loss, a dorsal lazy S-shaped incision was made over the dorsum of the hand, deepening through subcutaneous tissues until reaching the mass which was completely adherent to the extensor tendons. Difficult dissection of the mass was carried out to liberate the extensor tendons from the extensor retinaculum to the metacarpal heads. En bloc excision of the mass and a complete extensor tenosynovectomy were carried out. Subsequently, tissues were sent for pathology and the wound was closed in two layers. Figure 3 shows the different aspects of the surgical intervention.

Cultures had no growth after 48 hours. Pathology of the tissues specimens showed caseating granuloma, and a PCR test confirmed the diagnosis of *Mycobacterium tuberculosis* infection.

The patient was sent to an infectious disease specialist who treated him with a combination of ethambutol, isoniazid, rifampicin, and pyrazinamide for nine months.

**Figure 4:**
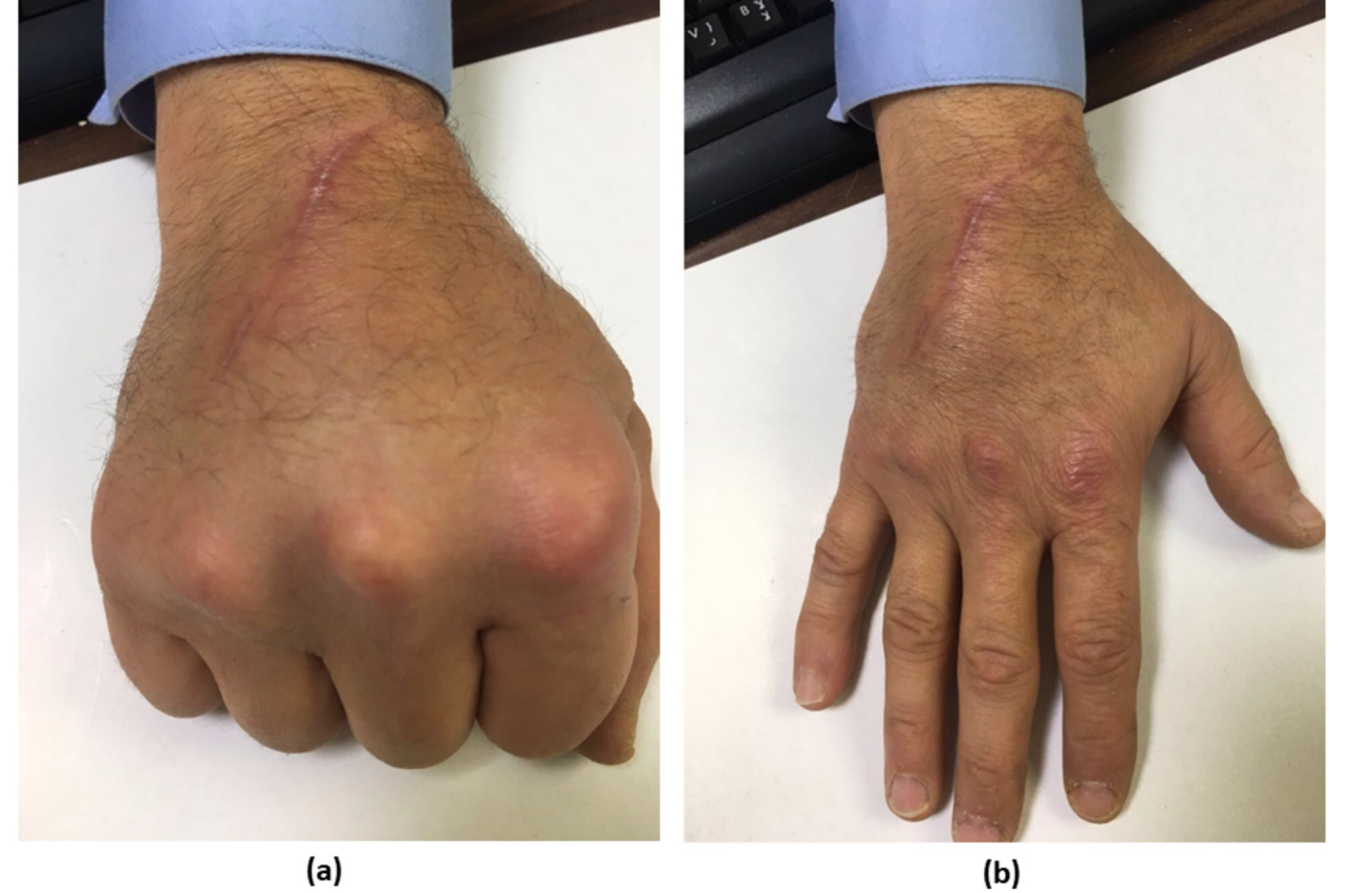
Full range of motion of fingers in flexion (a) and extension (b) after four months of the operation

Four months postoperatively, the patient presented to the clinic with complete recuperation of the inflammatory process at the surgical site, and a full recovery of the finger range of motion in flexion and extension (Figure 4).

Nine months postoperatively, the patient completed his anti-tuberculosis treatment regimen and was symptom-free without any sequelae.

## Discussion

TS is a medical term used to describe a wide variety of clinical conditions that involve inflammatory or non-inflammatory disruption of the tendon sheath [5]. It can be divided into three pathophysiologic groups: non-inflammatory, inflammatory, and infectious.

The non-inflammatory group, the most common type, is in turn divided into two subgroups. The first subgroup is stenosing TS mainly affecting the flexor tendons (mostly the ring finger followed by the thumb) [6]. It is present in 2% of the general population and 20% of the diabetic general population [7]. The second subgroup is DeQuervain TS involving the 1st extensor compartment of the wrist, which is present in 0.6 to 2.8 per 1000 person [8].

Another group is the inflammatory TS, in which etiological factors such as autoimmune immune disease, rheumatoid arthritis, trauma, microtrauma, or even idiopathic inflammation play an important role in the pathophysiological pathways of the disease. These types usually affect the dorsal extensor compartments of the wrist, such as extensor pollicis longus TS, 4th compartment TS, intersection syndrome, and extensor carpi ulnaris TS [5].

The last group is the infectious TS. Although no data exists about its incidence, it is very well known that the flexor tendons are more commonly involved than the extensor tendons [9]. The most common pathogen isolated in any infectious TS is *Staphylococcus aureus *[10]. *Mycobacterium tuberculosis* as a causative agent is uncommon [11]. Tuberculosis is a major infectious disease that mainly affects lung tissue, but may also infect other organs. The infection can be spread by two major routes: hematogenous or lymphatic [12].

Infected patients can be asymptomatic (latent tuberculosis) or develop active tuberculosis disease, which in turn can be primary extrapulmonary or combined pulmonary and extrapulmonary disease. Extrapulmonary tuberculosis represents about 14% of all tuberculosis cases [11]. The main affected organs include lymph nodes, pleura, bones and joints, brain and meninges, gastrointestinal organs, liver, genitourinary organs, peritoneum, and pericardium [13]. Among all cases of extrapulmonary tuberculosis, the musculoskeletal system is involved in about 1% to 3%, of which the hand as the main site of infection represents only 1% [14].

This extremely low incidence combined with the unusual involvement of the extensor tendon sheath makes the diagnosis of tuberculous TS of the extensor tendons of the hand a very unique and rare entity that was mentioned in very few case reports [3,4,11,15].

Risk factors include age >60 years, malnutrition, alcohol abuse, low socioeconomic status, history of or exposure to tuberculosis, immunosuppression from any cause, diabetes mellitus, and intake of corticosteroids [3].

On the tissue level, the tuberculous TS is classified into three stages. The first stage involves sheath thickening and serous exudation. The second stage is the proliferative stage showing granulomatous tissue that forms the rice bodies. The third and final stage involves the tissue necrosis, as the main issue that can be seen in this stage [11].

The challenge in diagnosing this rare disease stems from its nonspecific presentation. In fact, the typical presentation found in infectious flexor TS, such as the Kanavel signs, is absent in any infectious extensor TS [9].

The clinical signs of swelling along the tendon sheath can mimic many other conditions that affect the wrist such as De Quervain TS, granulomatous tophaceous gout, fungal infections, rheumatoid diseases (rheumatoid arthritis, sarcoidosis) and some types of tumors [16]. This can easily delay the diagnosis for a few months which could ultimately lead to tendon rupture [4]. Our case was misdiagnosed as infectious cellulitis delaying appropriate treatment for two months.

Laboratory data are nonspecific except for elevated CRP and ESR [15]. The diagnosis is aided by ultrasound which may reveal a thickening of the tendon sheath as well as fluid collection inside of it. MRI is a more sensitive and specific imaging modality and a paramount tool in guiding the diagnosis when the suspicion of tuberculous TS is high. It allows, in addition to the findings mentioned in the ultrasonography, the visualization of synovial proliferation, abscess formation, and the destruction of adjacent bone [17].

The diagnosis is confirmed by surgical excision, culture, and biopsy. Histopathological examination typically shows numerous caseating granulomas surrounded by epithelioid histiocytes and multinucleated giant cells. However, non-caseating granulomas can be seen in 27% of cases [18].

The mainstay of treatment consists of surgical debridement followed by 9 to 12 months of antibiotherapy with rifampicin, isoniazid, ethambutol, and pyrazinamide with good clinical outcomes reported in the literature. However, Aditya Jain et al. reported successful results in treating tuberculous TS of the extensor tendons with antibiotherapy alone. Our patient received a combination of ethambutol, isoniazid, rifampicin, and pyrazinamide for 9 months after surgical excision with favorable evolution [19].

## Conclusions

Tuberculous TS of the hand remains a rare pathology requiring a high index of suspicion. The unusual involvement of the extensor tendons, and the non-specificity of the clinical presentation, could challenge the physician and delay the diagnosis, which can be accurately made with the combined use of many tools such as ultrasonography, MRI, tissue culture, and histopathology. Although no consensus has been established on the treatment of this condition, surgical excision followed by 9 to 12 months of antibiotherapy with ethambutol, isoniazid, pyrazinamide, and rifampin yield good clinical outcomes.
